# Reversible junctional bigeminy in severe hyperkalemia: electrocardiographic and biochemical correlation: A case report

**DOI:** 10.1097/MD.0000000000046228

**Published:** 2025-11-21

**Authors:** Amrit Kooner, Robert Luong, Mutsumi Kioka

**Affiliations:** a Division of Pulmonary and Critical Care Medicine, Department of Internal Medicine, Kirk Kerkorian School of Medicine, University of Nevada, Las Vegas, NV.

**Keywords:** bigeminy, electrocardiogram, hyperkalemia, junctional escape, sinus node suppression

## Abstract

**Rationale:**

Severe hyperkalemia is classically associated with peaked T waves, PR prolongation, and QRS widening. However, sustained bigeminal rhythms resulting from junctional escape are exceptionally rare. Detailed correlations between electrocardiographic (ECG) changes and serum potassium levels remain scarce in the literature.

**Patient concerns:**

A 54-year-old man with end-stage renal disease on maintenance hemodialysis presented with hypotension and bradycardia.

**Diagnoses:**

ECG revealed a striking bigeminal rhythm characterized by alternating supraventricular and junctional escape complexes, an uncommon arrhythmic manifestation of hyperkalemia. Laboratory testing showed a serum potassium level of 7.5 mmol/L.

**Interventions:**

The patient underwent emergent hemodialysis for rapid potassium removal and received standard supportive management for hyperkalemia.

**Outcomes:**

Following hemodialysis, serum potassium normalized to 3.7 mmol/L, and the bigeminal rhythm resolved completely, with restoration of normal sinus rhythm. Serial ECGs demonstrated dynamic and reversible conduction changes in parallel with biochemical correction.

**Lessons:**

This case highlights a rare but reversible presentation of junctional bigeminy secondary to hyperkalemia. Recognition of this distinctive ECG pattern – correlated with paired serum potassium values – can facilitate timely diagnosis and prevent unnecessary interventions.

## 1. Introduction

Electrocardiographic manifestations of hyperkalemia are well recognized, typically including peaked T waves, PR interval prolongation, and QRS widening. Far less commonly, severe hyperkalemia can suppress sinoatrial node activity and give rise to bigeminal rhythms composed of alternating supraventricular and junctional escape complexes.^[1]^ Only a few cases have been described, and reports with clear before-and-after electrocardiograms paired with serum potassium values are extremely limited. We present a case of reversible junctional escape bigeminy in the setting of severe hyperkalemia, uniquely documented with biochemical correlation. Recognizing this rare but reversible arrhythmic manifestation is crucial for avoiding misdiagnosis and preventing unnecessary antiarrhythmic therapy or pacing interventions.

## 2. Case presentation

A 54-year-old man with a past medical history of end-stage renal disease on maintenance hemodialysis, hypertension, type 2 diabetes mellitus, dyslipidemia, hypothyroidism, and secondary hyperparathyroidism presented with fatigue and lightheadedness that began earlier in the day. He denied fever, chest pain, shortness of breath, or recent illness.

On arrival, he was found to be hypotensive (BP 82/42 mm Hg, MAP 54 mm Hg) and bradycardic (HR 44 bpm). Oxygen saturation was 94% on 2 L/min nasal cannula. Laboratory evaluation revealed severe hyperkalemia with a serum potassium of 7.5 mmol/L. Other labs included sodium 134 mmol/L, bicarbonate 19 mmol/L, BUN 99 mg/dL, creatinine 11.63 mg/dL, lactate 2.32 mmol/L, and hemoglobin 9.3 g/dL. Arterial blood gas showed a mild acidemia (pH 7.304) with a bicarbonate of 20.9 mmol/L.

Initial management included nebulized albuterol (10mg), intravenous calcium gluconate (1 g), and dextrose 10% (250 mL) followed by 5 units of intravenous regular insulin, administered prior to emergent hemodialysis.

**Figure 1.**
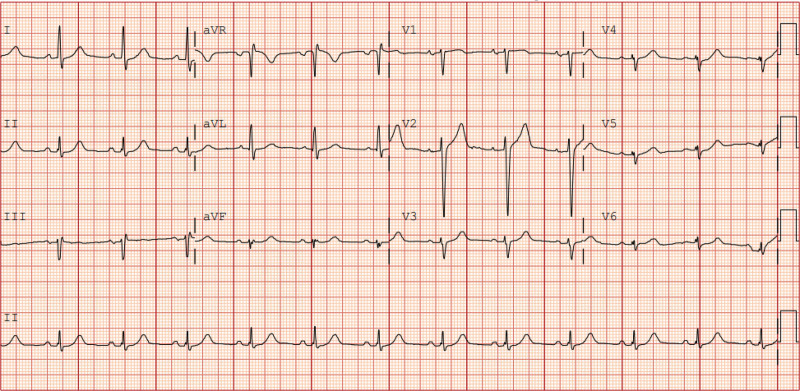
Baseline electrocardiogram. Five months prior to presentation, when serum potassium was 3.7 mmol/L, showing normal sinus rhythm without conduction or repolarization abnormalities.

**Figure 2.**
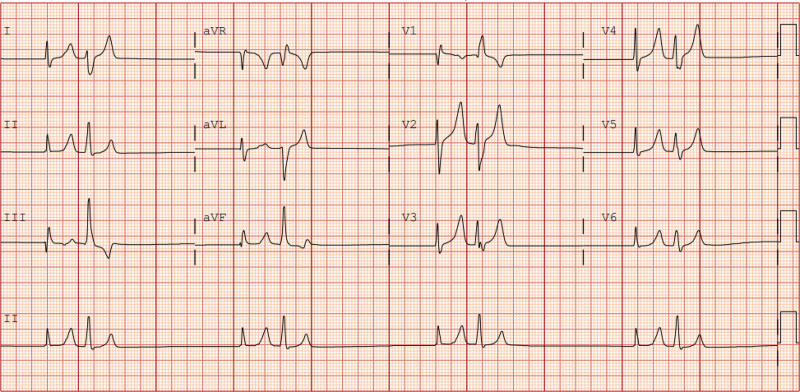
Junctional escape bigeminy during severe hyperkalemia. On presentation with serum potassium of 7.5 mmol/L, demonstrating alternating supraventricular and junctional escape complexes consistent with a bigeminy.

**Figure 3.**
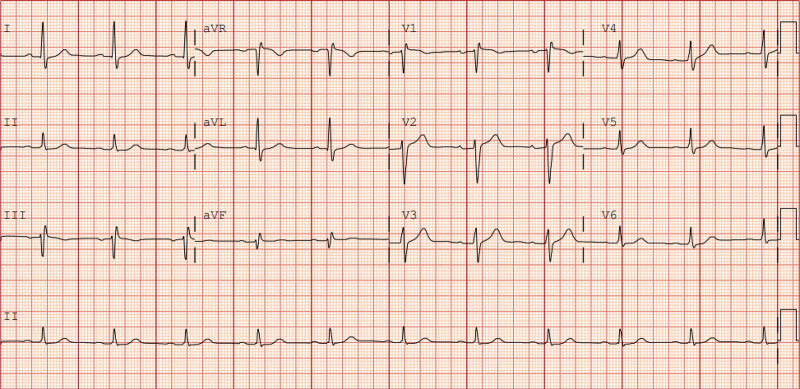
Resolution after potassium correction. Following hemodialysis, when serum potassium normalized to 3.7 mmol/L, showing normal sinus rhythm and resolution of bigeminy.

At baseline, 5 months prior, his potassium was 3.7 mmol/L, and an ECG at that time showed a normal sinus rhythm (Fig. 1). On the current presentation with serum potassium of 7.5 mmol/L, the ECG demonstrated a striking bigeminal pattern of alternating supraventricular complexes and junctional complexes in a bigeminal pattern (Fig. 2). Following hemodialysis, his potassium levels normalized to 3.7 mmol/L, and a repeat ECG showed conversion back to normal sinus rhythm. However, the P wave was slightly widened, indicating delayed conduction from the SA node to the atria. PR interval prolongation suggested residual impairment of the atrioventricular nodal action potential. An incomplete right bundle branch block pattern was present, suggesting persistent conduction disturbance predominantly in the right ventricle. (Fig. 3).

Serial high-sensitivity troponin I levels were elevated and mildly dynamic, measured at 487 ng/L, peaking at 525 ng/L, then decreasing to 394 ng/L – consistent with type 2 myocardial injury (demand ischemia) secondary to hemodynamic compromise.

Cardiology was consulted, and the patient was admitted to the intensive care unit for management of hyperkalemia-induced shock. He reported a similar episode in the past after missing a dialysis session; however, in this case, he had received dialysis 2 days prior and had not missed any treatments. He admitted non-adherence to his potassium-binding medication for the past week but denied any dietary indiscretion or recent illness.

His home medications included amlodipine, atorvastatin, carvedilol, fenofibrate, insulin lispro, levothyroxine, metoprolol tartrate, and nifedipine XL. He had an arteriovenous fistula for dialysis access. There was no significant family history, and he denied tobacco, alcohol, or illicit drug use. He reported no known drug allergies.

## 3. Discussion

This case highlights a rare but reversible manifestation of severe hyperkalemia: junctional escape bigeminy. While conduction abnormalities in hyperkalemia are well described, sustained bigeminal rhythms consisting of alternating sinus and junctional escape complexes are seldom reported. In our patient, the arrhythmia resolved completely following normalization of serum potassium, confirming a direct causal relationship.^[2]^

To better understand this presentation – particularly the widened QRS and peaked T waves – it is helpful to review the effects of hyperkalemia on cardiac action potentials, focusing on phases 4, 0, and 3.

### 3.1. Pacemaker action potential (Fig. 4A)

Phase 4: Spontaneous diastolic depolarization: Initiated by the hyperpolarization-activated “funny” Na^+^ current, followed by transient T-type Ca^2+^ channel opening. This gradual depolarization underlies SA node automaticity and heart rate control.^[3]^
Phase 0: Once threshold is reached, L-type Ca^2+^ channel open, producing the upstroke. Because pacemaker cells lack fast Na^+^ channels, the upstroke is slower than in atrial or ventricular myocytes.^[4]^
Phase 3: Inactivation of L-type Ca^2+^ channels with simultaneous activation of delayed rectifier K^+^ currents returns the membrane to its most negative potential, completing the cycle.^[4]^

**Figure 4.**
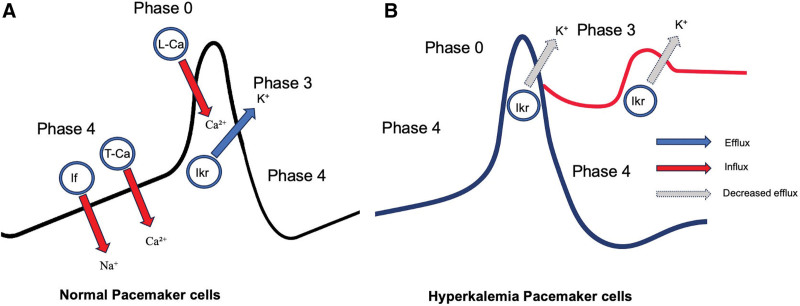
(A and B) Alterations in pacemaker cell action potential during hyperkalemia. (A) The normal pacemaker action potential is driven by gradual Phase 4 diastolic depolarization, primarily due to inward (“funny” current) via If channel and T-type calcium influx (T-Ca), followed by Phase 0 upstroke mediated by L-type calcium channels (L-Ca). Phase 3 repolarization is achieved via potassium efflux through delayed rectifier channels (Ikr). (B) In hyperkalemia, the reduced potassium efflux gradient slows repolarization and blunts membrane potential changes, leading to a flattened or distorted action potential. These changes may contribute to AV nodal suppression, junctional rhythms, and escape–capture patterns observed in hyperkalemia-associated ECGs. (Adapted with permission from educational materials by Dr Riku Arai, Nihon University School of Medicine.)

### 3.2. Junctional escape in a bigeminal pattern (Fig. 4B)

Hyperkalemia partially depolarizes pacemaker cells, slowing phase 4 and suppressing SA node firing. This allows AV junctional pacemakers to “escape” intermittently, producing alternating sinus and junctional complexes. Unlike ventricular bigeminy, these beats are nonpremature and originate from the AV junction. Resolution after potassium normalization confirms the reversible, electrolyte-driven mechanism.^[5]^

### 3.3. Ventricular myocyte action potential (Fig. 5)

Phase 4: Elevated extracellular potassium reduces the K^+^ gradient, partially depolarizing the resting membrane potential. Outward current through Ik1 diminishes, destabilizing the membrane and increasing excitability.^[6]^
Phase 0: Partial depolarization inactivates voltage-gated Na^+^ channels, slowing the upstroke velocity and impairing ventricular conduction. This manifests as QRS widening.^[7]^
Phase 3: At more positive potentials, enhanced outward K^+^ currents (Ikr/Iks) accelerate repolarization, generating the characteristic peaked T waves.^[7]^

**Figure 5.**
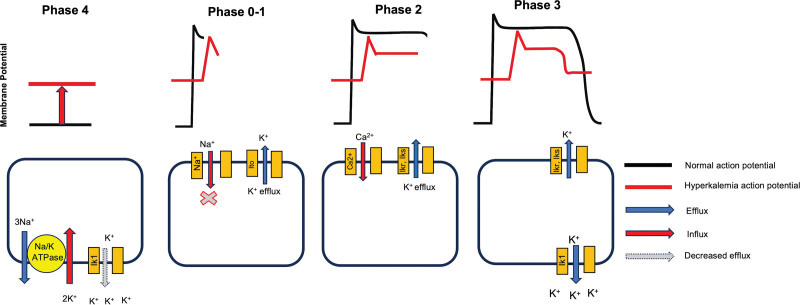
Alterations in ventricular myocyte action potential during hyperkalemia. In Phase 4, elevated extracellular potassium reduces the transmembrane potassium gradient, diminishing the outward driving force via the inward rectifier current (Ik1) and partially depolarizing the resting membrane potential. In Phase 0, this depolarization causes steady-state inactivation of voltage-gated sodium channels, resulting in a slower upstroke velocity and QRS widening on ECG. In Phase 3, the more positive membrane potentials increase the electrochemical driving force for K^+^ efflux through delayed rectifier (IKr/Iks) and Ik1 channels, thereby accelerating repolarization and producing the characteristic peaked T waves. These electrophysiological alterations account for the hallmark ECG manifestations of hyperkalemia. (Adapted with permission from educational materials by Dr Riku Arai, Nihon University School of Medicine.)

### 3.4. Previous case reports

Case reports have described a spectrum of hyperkalemia-induced conduction disturbances presenting with various forms of bigeminy, depending on potassium level and the underlying conduction substrate. For example, at moderate hyperkalemia (~5.8 mEq/L), sinus node suppression produced junctional escape rhythm with atrial bigeminy, which resolved after hemodialysis.^[5]^ At higher levels (~7.0 mEq/L), escape–capture bigeminy was observed, resolving with intravenous calcium and insulin-glucose therapy.^[2]^ Even mild hyperkalemia has been associated with escape–echo bigeminy, indicating that small shifts in membrane excitability can unmask latent conduction abnormalities^[8]^ (Table 1).

**Table 1:** Comparative summary of case reports on hyperkalemia-induced bigeminy patterns.

Feature	Case 1: Moderate hyperkalemia	Case 2: Severe hyperkalemia	Case 3: Mild hyperkalemia
**Serum potassium**	~5.8 mEq/L	~7.0 mEq/L	Mildly elevated
**ECG findings**	Junctional rhythm with atrial bigeminy	Escape–capture bigeminy	Escape–echo bigeminy
**Primary mechanism**	SA node suppression with AV node takeover	Alternating junctional escape and sinus capture	Echo beat following junctional escape
**Treatment**	Hemodialysis	IV calcium, insulin, dextrose	Supportive care only
**Outcome**	Restoration of normal sinus rhythm	Full resolution of bigeminy	No pacing required; rhythm normalized

Collectively, these reports highlight the electrophysiological spectrum of hyperkalemia, ranging from sinus node suppression to enhanced junctional or ventricular automaticity. In all cases, timely correction restored sinus rhythm without pacing.

Our case expands this spectrum by documenting a reversible sinus-junctional escape bigeminy, supported by before-and-after ECGs and serum potassium values, underscoring the dynamic interplay between electrolyte derangement and cardiac conduction.

#### Comparison with other metabolic arrhythmias

Hyperkalemia diminishes sinus node automaticity and slows AV conduction, predisposing to junctional escapes and a bigeminal pattern that resolves promptly once potassium is corrected. By contrast, hypokalemia promotes ventricular ectopy and risk of torsades de pointes – typically with U waves and QT prolongation – while hypomagnesemia lowers the threshold for polymorphic VT. Calcium derangements chiefly influence QT duration (shorter in hypercalcemia and longer in hypocalcemia). Recognizing these metabolic signatures helps avoid unnecessary antiarrhythmics or pacing and directs rapid, electrolyte-targeted therapy. Electrolytes and outcomes: Beyond their mechanistic role in triggering arrhythmias, electrolyte imbalances themselves are linked to adverse outcomes, and correcting them is a key prognostic intervention in acute care. A concise review highlights how disturbances in K^+^/Mg^2+^/Ca^2+^ predispose to malignant arrhythmias and worsen prognosis, underscoring the need for protocolized monitoring and replacement.^[9]^

#### Follow-up and prevention

After stabilization, we advise structured outpatient monitoring of serum potassium and renal/renin–aldosterone parameters, medication reconciliation – including, angiotensin II receptor blockers (ARB), mineralocorticoid receptor antagonists, potassium-sparing diuretics, and nonsteroidal anti-inflammatory drugs (NSAIDs), dietary counseling, and dialysis-prescription review when applicable. Observational myocardial infarction cohorts demonstrate a U-shaped relationship between potassium and mortality, with the lowest risk around 4.0 to 4.5mmol/L, supporting maintenance of potassium in the mid-normal range for both short- and long-term follow-up.^[10]^

#### Other factors that shift electrolytes

Beyond renal failure and medications, tissue injury can acutely raise potassium. Rhabdomyolysis after targeted temperature management (therapeutic hypothermia) has been reported and can precipitate hyperkalemia and acute kidney injury (AKI), underscoring the need for serial electrolytes after rewarming.^[11]^

#### Risk stratification with IMRS

The Intermountain Risk Score (IMRS) – a validated mortality score composed of routine CBC and basic metabolic panel elements (including potassium, sodium, bicarbonate, calcium, creatinine, glucose) plus age/sex – has shown prognostic value for short- and long-term mortality in STEMI, and even among patients with STEMI complicated by cardiogenic shock. While not specific to hyperkalemia-induced junctional rhythms, the inclusion of potassium within IMRS underlines its prognostic weight and supports maintaining mid-normal K^+^ and serial electrolyte surveillance during follow-up.^[12,13]^

## 4. Limitation

The primary limitation of this report is that the arrhythmia may have been influenced not only by hyperkalemia but also by additional factors such as medications, hypoxia, or acid–base disturbances, which cannot be fully excluded. Nonetheless, the temporal resolution of the bigeminal pattern following potassium correction strongly supports hyperkalemia as the principal driver.

## 5. Conclusion

This case illustrates a rare but reversible manifestation of severe hyperkalemia: junctional escape bigeminy due to sinus node suppression. The availability of paired before-and-after ECGs with corresponding serum potassium values provides unique evidence for a direct causal relationship. Awareness of this presentation is important for clinicians, as it can prevent misdiagnosis and avoid unnecessary antiarrhythmic or pacing interventions. Prompt recognition and potassium correction remain the cornerstone of management in such cases.

## Acknowledgments

The author thanks Dr Riku Arai of Nihon University School of Medicine for granting permission to use the figure and for providing valuable advice.

## Author contributions

**Conceptualization:** Mutsumi Kioka.

**Investigation:** Mutsumi Kioka, Amrit Kooner, Robert Luong.

**Project administration:** Mutsumi Kioka.

**Supervision:** Mutsumi Kioka.

**Validation:** Mutsumi Kioka.

**Visualization:** Mutsumi Kioka.

**Writing – original draft:** Mutsumi Kioka, Amrit Kooner, Robert Luong.

**Writing – review & editing:** Mutsumi Kioka.
